# Diagnostic and prognostic value of ABC transporter family member ABCG1 gene in clear cell renal cell carcinoma

**DOI:** 10.1080/19336950.2021.1909301

**Published:** 2021-04-07

**Authors:** Fucheng Meng, Yafei Xiao, Longxiang Xie, Qiao Liu, Keli Qian

**Affiliations:** a Department of Infection Control, The First Affiliated Hospital of Chongqing Medical University, Chongqing, China; b Department of Institute of Biomedical Informatics, Cell Signal Transduction Laboratory, Bioinformatics Center, Henan Provincial Engineering Center for Tumor Molecular Medicine, School of Basic Medical Sciences, Henan University, Kaifeng, China; c Department of Pediatric Dentistry, Stomatological Hospital of Chongqing Medical University, Chongqing, China

**Keywords:** ABCG1, ccRCC, diagnosis, prognosis, cholesterol efflux

## Abstract

As the most common histologic subtype of renal cancer, clear cell renal cell carcinoma (ccRCC) poses a serious threat to public health. However, there are no specific molecular-targeted drugs for ccRCC at present. Human ATP-binding cassette (ABC) transporter family plays an important role in homeostasis maintenance. This study aimed to evaluate the potential diagnostic value of *ABC* genes in ccRCC. A total of 952 samples of ccRCC patients (707) and controls (245) from three different datasets were included for analysis. Receiver operating characteristic analysis and t-test were used to analyze the differential expression of *ABC* genes in ccRCC patients and control samples at mRNA level during screening and validations. The Cancer Genome Atlas (TCGA-ccRCC) dataset was utilized to investigate the correlation between ABC genes expression and prognostic value in ccRCC. We then investigated the interactions between *ABCG1* and proteins in the Comparative Toxicogenomics Database (CTD). Finally, we found that ATP-binding cassette transporter G member 1 (*ABCG1*) was over-expressed in ccRCC patients compared with healthy samples at mRNA level. Cox regression analysis and Kaplan–Meier analysis showed that ccRCC patients with high *ABCG1* expression had better overall survival (OS) than those patients with low expression (hazard ratio (HR) = 0.662, *p* = 0.007). This study demonstrated that *ABCG1* is a potential diagnostic and prognostic biomarker in ccRCC and discussed the molecular mechanisms underlying the relationship between ccRCC and *ABCG1*, which might provide guidance for better management and treatment of ccRCC in the future.

## Introduction

Renal cell carcinoma (RCC) has become one of the most common malignant tumors in urology and accounts for 85% of primary renal cancer. It was estimated that almost 403,262 (2.2%) new cases of kidney cancer and 175,098 (1.8%) deaths worldwide occurred in 2018 [1]. Besides, the global morbidity and mortality of RCC are increasing by approximately 2–3% per decade [2]. Clear cell renal cell carcinoma (ccRCC) is the most common pathological type of RCC in adults. Surgery is recommended as the preferred option in local ccRCC [3], with five-year survival at more than 90% [4]. Then, due to the absence of obvious clinical symptoms at the early stage, cancer metastasis has occurred in 25–30% patients at the time of initial diagnosis of ccRCC [5]. Although there has been a significant progress in the management of advanced ccRCC, with improved knowledge of disease and the application of targeted drugs. Five-year survival drops to 12% for patients with metastatic ccRCC [4]. Therefore, identification and validation of biomarkers will be crucial for optimizing the management of ccRCC. In recent years, many molecular biomarkers for ccRCC have been discovered. C1q/tumor necrosis factor (C1QTNF) [6] and six-snoRNA (small nucleolar RNA) signature (*SNORA2*, *SNORD12B*, *SNORA59B*, *SNORA70B*, *SNORD93*, and *SNORD116-2*) [7] could serve as an independent diagnostic and prognostic indicator for ccRCC. Because of the lack of precise and effective molecular targets for the therapy of ccRCC, it is still important to explore new molecular markers or therapeutic targets for the diagnosis and prognosis [8].

Human ATP-binding cassette (ABC) transporter family contains 49 members that are divided into eight subfamilies [9]. ABC transporter family is a widespread membrane-bound protein, which is mainly distributed in liver, intestine, blood–brain barrier, blood–testosterone barrier, placenta, and kidney. ABC protein can transport various endogenous substrates, including inorganic anions, metal ions, peptides, amino acids, sugars, hydrophobic, and metabolites [10]. Abnormal changes of the ABC genes can lead to multiple diseases, such as cystic fibrosis and disorder of cholesterol metabolism [11]. It has been demonstrated that cholesterol metabolism disorders are related to various cancers, and the cholesterol level in cancer cells elevates obviously compared with normal tissues [12,13].

Recently, several studies had explored the role of 10 ABC family members in ccRCC. *ABCA1* [14] and *ABCD1* [15] were related to the occurrence and development of tumors. *ABCA13* [16,17], *ABCB1* [18], *ABCC1* [19], and *ABCC2* [20,21] were associated with drug resistance and treatment of tumors. *ABCB2* and *ABCB3* were found to be involved in tumor immune evasion [21,22]. *ABCG2* was correlated with tumor progression, prognosis [23] and drug resistance [18], and *ABCB10* was associated with tumor progression as well as prognosis [24]. In this study, we mainly focused on the expression of ABC genes in multiple datasets to assess their diagnostic and prognostic value in ccRCC.

## Material and methods

**Figure 1.**
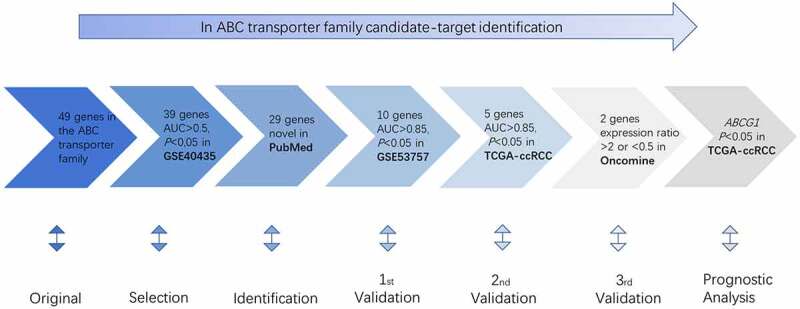
Procedure for the selection of the potential biomarker in ccRCC

Gene expression of ABC family members in 952 samples from three independent public datasets (GSE40435 dataset, GSE53757 dataset, and TCGA-ccRCC dataset) was analyzed by screening and verification. Transcriptional expression of ABC genes from the Oncomine database (http://www.oncomine.org) was also investigated. Then, a prognostic analysis of the validated gene was conducted on TCGA-ccRCC dataset from UCSC Xena (https://xenabrowser.net) (Figure 1). The procedure was similar to the previous studies [25].

### Screening of ABC genes in the Gene Expression Omnibus (GEO) database – ccRCC vs normal samples

GSE40435 dataset containing 202 samples (101 ccRCC patients and 101 healthy controls) and the corresponding probe set GPL10558 was obtained from NCBI-GEO database (https://www.ncbi.nlm.nih.gov/geo/query/). T-test and receiver operating characteristic (ROC) analysis were carried out for analyzing the difference in gene expression on 202 samples. *p* < 0.05 of t-test and the area under curve (AUC)>0.5 of ROC analysis were considered to be significant statistically.

### Identification of genes that have not been reported in ccRCC

The genes selected in the screening stage were searched for ccRCC-related research in PubMed (https://www.ncbi.nlm.nih.gov/) on 5 December 2019. Our specific advanced search terms included “Renal clear cell carcinoma” OR “Clear cell renal cell carcinoma” OR “Kidney clear cell carcinoma” OR “KIRC” OR “ccRCC.” The genes that had not been studied before were considered new genes and then selected for the following validation.

### Three rounds of validation

The first round of validation was performed by investigating the expression of the identified genes in the GSE53757 dataset from the NCBI-GEO database. The ROC analysis and t-test were carried out on 144 samples (72 ccRCC patients and 72 controls). Genes with *p* < 0.05 and AUC > 0.85 were selected for the following validation. The second round of validation was performed on TCGA-ccRCC dataset. ROC analysis and t-test were performed on 606 samples (72 healthy samples and 534 ccRCC patients). Genes with *p* < 0.05 and AUC > 0.85 were considered validated. The third round of validation was performed by analyzing transcriptional expression of ABC genes in 20 different tumors in the Oncomine database. The thresholds were as follows: *p* value: 0.05; multiple: 2; genetic rank: top 10%; data type: mRNA. Then, we found out the corresponding study on clear cell carcinoma of the kidney. The genes showing an expression ratio >2 or <0.5 were considered effective to be validated.

### Prognostic analysis

To evaluate the prognostic value of the clinical characteristics in ccRCC patients, we analyzed the relationship between *ABCG1* expression and clinical–pathological parameters including carcinoma in situ, expression, age, gender, survival outcome, overall survival (OS), stage, recurrence, survival after recurrence time (RFS), and smoking history from TCGA-ccRCC dataset. Five hundred and thirty-two patients with clinicopathologic information were equally divided into two groups on the basis of the gene expression. Univariate Cox regression analysis was carried out to find independent variables. Multivariate Cox regression analysis was performed for the parameters with *p* < 0.2 in univariate Cox regression analysis to assess the prognostic value.

### Statistical analysis

ROC analysis and t-test were carried out with GraphPad 8.0 software during screening and validation. In the prognostic analysis step, univariate and multivariate analyses were performed on SPSS19.0. A chi-square test was performed and the OS curve of validated genes was also constructed using GraphPad 8.0 software.

## Results

### Screening and validation

The expression data of 49 *ABC* genes were collected from the GSE40435 dataset and samples were divided into two groups (patient group and control group). ROC analysis and t-test were used to evaluate the ability to discriminate ccRCC patients from control samples. The results showed that 39 genes have statistical significance with AUC > 0.5 and *p* < 0.05 (Table 1). A PubMed search was conducted on 5 December 2019. We found 29 genes that had not been reported to be associated with ccRCC (Table 1).

**Table 1.** ROC analysis and t-test of ABC transporter family based on GSE40435 dataset

**NO.**	**Gene**	**Expression (**ccRCC)	**Expression (**normal)	**ccRCC/normal**		**AUC**	***p***	**Gene and ccRCC** **co-occurrence**	
1	*ABCA1*	10.860	9.804	1.108	↑	0.961	<0.0001		≥1
2	*ABCA2*^a^	7.135	7.199	0.991	↓	0.604	0.0074		0
3	***ABCA3***	8.022	7.494	1.070	↑	0.901	<0.0001		0
4	***ABCA4***	6.769	7.160	0.945	↓	0.883	<0.0001		0
5	***ABCA5***	6.918	7.027	0.984	↓	0.742	<0.0001		0
6	***ABCA6***	7.015	7.332	0.957	↓	0.845	<0.0001		0
7	***ABCA8***	7.059	7.683	0.919	↓	0.946	<0.0001		0
8	***ABCA9***	6.925	7.227	0.958	↓	0.929	<0.0001		0
9	***ABCA10***	6.776	6.808	0.995	↓	0.646	<0.0001		0
10	***ABCA12***	6.728	6.582	1.022	↑	0.883	<0.0001		0
11	*ABCA13*	6.495	6.614	0.982	↓	0.740	<0.0001		≥1
12	*ABCB1*	7.870	9.453	0.833	↓	0.982	<0.0001		≥1
13	*ABCB2(TAP1)*	11.140	8.970	1.242	↑	0.999	<0.0001		≥1
14	*ABCB3(TAP2)*	6.818	6.662	1.023	↑	0.774	<0.0001		≥1
15	***ABCB4***	7.096	6.814	1.041	↑	0.744	<0.0001		0
16	***ABCB6***	8.734	8.257	1.058	↑	0.809	<0.0001		0
17	***ABCB8***	6.884	6.995	0.984	↓	0.739	<0.0001		0
18	***ABCB9***	7.292	7.395	0.986	↓	0.735	<0.0001		0
19	*ABCB10*	8.416	8.183	1.028	↑	0.762	<0.0001		≥1
20	*ABCC1*	6.973	6.823	1.022	↑	0.881	<0.0001		≥1
21	*ABCC2*	7.694	8.189	0.940	↓	0.738	<0.0001		≥1
22	***ABCC3***	8.847	8.059	1.098	↑	0.918	<0.0001		0
23	***ABCC4***	8.715	8.846	0.985	↓	0.596	0.0238		0
24	***ABCC5***	7.652	7.821	0.978	↓	0.737	<0.0001		0
25	***ABCC6***	7.063	7.610	0.928	↓	0.911	<0.0001		0
26	***ABCC8***	6.607	6.688	0.988	↓	0.755	<0.0001		0
27	***ABCC9***	7.021	6.891	1.019	↑	0.752	<0.0001		0
28	***ABCC10***	7.145	7.264	0.984	↓	0.687	<0.0001		0
29	***ABCC11***	6.722	6.707	1.002	↑	0.579	0.0197		0
30	***ABCC13***	6.727	6.740	0.998	↓	0.593	0.02		0
31	*ABCD1*	7.406	7.179	1.032	↑	0.756	<0.0001		≥1
32	***ABCD3***	7.983	8.618	0.926	↓	0.902	<0.0001		0
33	***ABCE1***	8.864	8.598	1.031	↑	0.842	<0.0001		0
34	***ABCF1***	9.295	9.626	0.966	↓	0.877	<0.0001		0
35	***ABCF2***	7.334	7.283	1.007	↑	0.587	0.0026		0
36	***ABCF3***	7.363	7.502	0.981	↓	0.736	<0.0001		0
37	***ABCG1***	7.591	7.139	1.063	↑	0.990	<0.0001		0
38	*ABCG2*	7.234	7.088	1.021	↑	0.557	0.0036		≥1
39	***ABCG4***	7.032	7.074	0.994	↓	0.602	0.0035		0

^a^Novel genes were marked in bold.

### First round of validation

The 29 genes obtained from the above steps were then validated in the GSE53757 dataset. The 10 genes showing AUC > 0.85 and *p* < 0.05 were allowed to enter the second round of validation, namely *ABCA3*, *ABCA8*, *ABCA9*, *ABCA12*, *ABCC3*, *ABCC6*, *ABCC8*, *ABCD3*, *ABCF1*, and *ABCG1* (Table S1). Notably, *ABCA12* showed the most significant difference in expression in ccRCC patients vs healthy samples, and the *ABCG1* gene with the highest AUC value.

### Second round of validation

**Figure 2.**
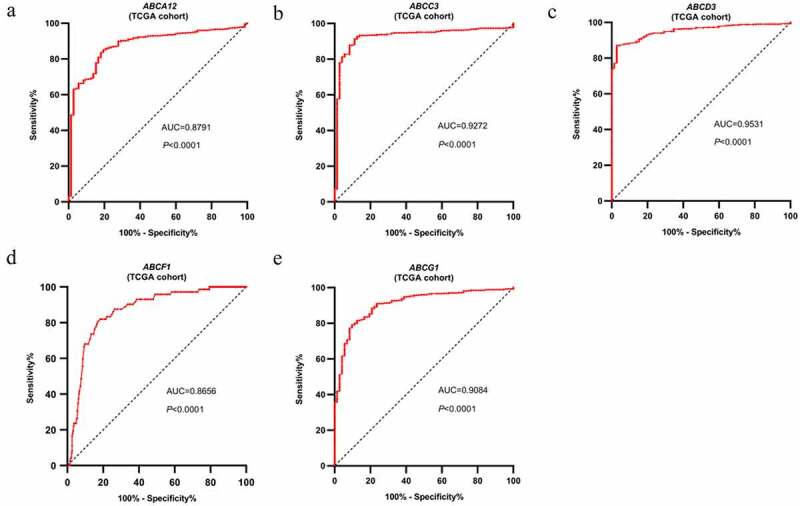
ROC analysis of the expression data for diagnostic assessment of five genes according to TCGA database. AUC statistics are used to evaluate the capacity to discriminate ccRCC samples from normal controls with specificity and sensitivity

Ten genes selected from the first-round validation were analyzed on the TCGA-ccRCC dataset for the second round of validation. Five genes with AUC > 0.85 and *p* < 0.05 were statistically significant, namely, *ABAC12*, *ABCC3*, *ABCD3*, *ABCF1*, and *ABCG1* (Table 2). ROC analysis was carried out to assess the diagnostic value of these five genes. The AUC values of the five genes indicated that they could identify ccRCC patients from normal samples effectively and independently (Figure 2).

**Table 2.** T-test and ROC analysis of ABC transporter family members based on the TCGA-ccRCC dataset

**No.**	**Gene**	**Expression** (ccRCC)	**Expression** (normal)	**ccRCC/normal**	**AUC**	***p***
1	*ABCA12*	6.50	3.42	1.90	0.88	<0.0001
2	*ABCC3*	11.56	9.14	1.26	0.93	<0.0001
3	*ABCD3*	10.43	11.49	0.91	0.95	<0.0001
4	*ABCF1*	10.24	10.67	0.96	0.87	<0.0001
5	*ABCG1*	10.44	9.09	1.15	0.91	<0.0001

### Third round of validation

Transcriptional expression of the above five genes was verified in the Oncomine database. The expression of *ABCC3*, *ABCF1*, and *ABCG1* in 20 types of cancers is shown in Figure S1. *ABCA12* and *ABCD3* had no available data. There are eight, five, and eight datasets of *ABCC3*, *ABCF1*, and *ABCG1* for renal cancer, respectively. However, there is no clear cell renal cell carcinoma vs normal in the five datasets of *ABCF1.*The result showed that *ABCG1* was highly overexpressed in all datasets (Table S2, Table S3).

### Prognostic analysis

**Figure 3.**
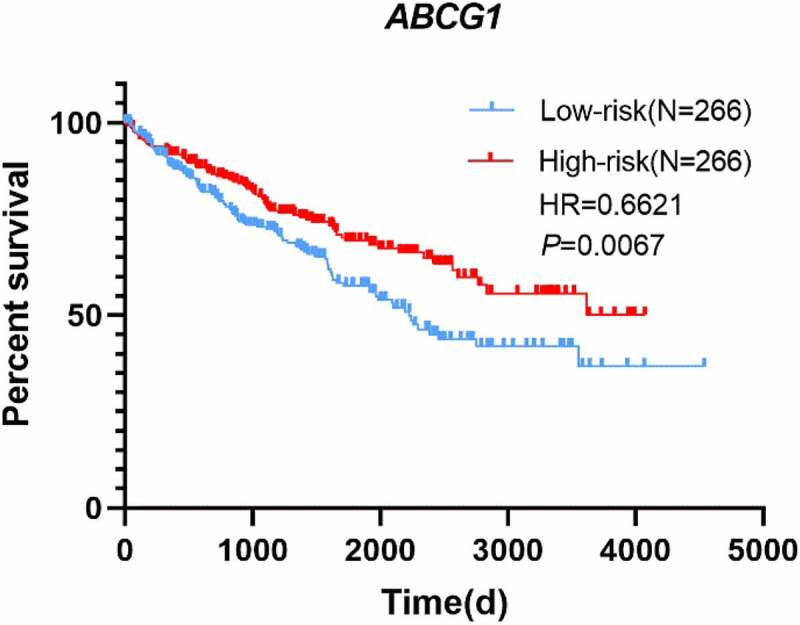
Kaplan–Meier survival curve of *ABCG1* mRNA expression in ccRCC patients. Survival curves suggested that patients with decreased *ABCG1* mRNA level significantly correlated with poorer OS (*p* = 0.0067)

To explore the prognostic value of *ABCG1* expression in the TCGA-ccRCC dataset, Cox regression analysis and Kaplan–Meier analysis were performed. The relationship between *ABCG1* expression and clinical characteristics in ccRCC is shown in Table 3. We found significant differences in living status between the high-expression group and the low-expression group (*p* = 0.01), but there was no statistical difference in gender, clinical stage, smoking history, and recurrence history between the two groups. Univariate Cox regression analysis showed that age, clinical stage, and *ABCG1* expression were associated with OS (Table 4). Meanwhile, multivariate Cox regression analysis demonstrated that *ABCG1* expression might be an independent prognostic factor for ccRCC patients. Kaplan–Meier analysis also showed that ccRCC patients with high *ABCG1* expression was significantly associated with better OS than those patients with low *ABCG1* expression (*p* = 0.0067, hazard ratio (HR) = 0.6621) (Figure 3).

**Table 3.** Chi-square test of clinicopathologic parameters and *ABCG1* mRNA expression in the TCGA-ccRCC cohort

Parameters	Group	*ABCG1* mRNA expression			
		High (n = 266)	Low (n = 266)	χ2	p
Age (Mean + SD)		60.80 ± 12.77	60.36 ± 12.03		
Gender	Female	82	104	4.001	0.056
	Male	184	162		
Clinical stage	I/II	164	188	4.872	0.034
	III/IV	101	77		
	Discrepancy	1	1		
Recurrence status	No	68	53	0.325	0.655
	Yes	15	9		
	Null	183	204		
Smoking history	1	29	16	2.147	0.143
	2/3/4/5	20	21		
	Null	229	217		
Living status	Living	193	164	7.161	0.010
	Dead	73	102

**Table 4.** Univariate and multivariate Cox regression analysis of clinical pathologic features according to the TCGA-ccRCC dataset

Parameters	Univariate analysis				Multivariate analysis		
	HR	95%CI	p		HR	95%CI	p
Age							
≥60 vs <60	1.808	1.321–2.478		0.000	1.563	1.140–2.144	0.006
Gender							
Female vs male	1.060	0.779–1.442		0.710	-	-	-
Clinical stage							
III/IV vs I/II	3.838	2.799–5.292		0.000	3.675	2.676–5.046	0.000
Smoking history							
2/3/4/5 vs 1	0.778	0.254–2.383		0.661	-	-	-
*ABCG1* expression							
High vs low	0.665	0.492–0.898		0.008	0.662	0.490–0.895	0.007

## Discussion

Many studies have demonstrated that ABC family genes play important roles in the maintaining cellular environment [26,27], cholesterol metabolism [9,10,28–31], disease occurrence [32–34], and tumor resistance [35,36]. ABC transporter genes could promote drug efflux and enhance chemical resistance of cancer cells [37]. Mutations in the *ABC* genes could affect the phenotypes of cancer cells such as proliferation, differentiation, migration, and invasion [38]. *ABCG1* is involved in lipid balance and cholesterol efflux from macrophages [10]. *ABCG1* is also able to transport sterols, which can regulate the expression of macrophage inflammatory cytokines, chemokines, and lymphocyte proliferation response [28]. *ABCG1* was found to be a potential biomarker for lung cancer [39,40], head and neck squamous cell carcinoma [41], and prostate cancer [42]. However, it has not been studied in ccRCC. After screening, identification, and three rounds of verification, *ABCG1* was selected from 49 ABC transporter genes. We first showed that *ABCG1* has the diagnostic and prognostic value for ccRCC patients.

Metabolic change is the main feature of tumors [43], and ccRCC is also considered as metabolic disease [44], which is characterized by the accumulation of cholesterol, cholesterol esters, other neutral lipids, and glycogen [45]. The total cholesterol content in the ccRCC tissues is eight times higher than that of the normal kidney, and the esterified cholesterol content is 35 times higher than that of the normal kidney [46]. The abnormalities in cholesterol metabolism in ccRCC cells may affect the physiological and biochemical functions of cells and produce pathological changes. Many studies have shown that serum cholesterol levels are associated with ccRCC invasion and prognosis [47–49]. Patients with low preoperative cholesterol levels have lower OS than patients with high cholesterol levels [47], and cholesterol metabolism may be involved in ccRCC metastasis [50]. The function of the ABCG1 gene is mainly related to cell cholesterol outflow [10], which indicates that *ABCG1* may play an important role in the tumorigenesis and progress of ccRCC.

Our study showed that *ABCG1* was over-expressed among patients with ccRCC compared with normal people (Table 1–2, Table S1, Figure 3). It may be hypothesized that when normal cells mutate into cancer cells, the energy demand increases, which activates a certain cholesterol transport mechanism and begins to take cholesterol from the outside. The decrease in serum cholesterol levels is correlated with the uptake of low-density lipoprotein in serum by tumor cells [49]. Yang et al. found that the accumulation of cholesterol is one of the characteristics of ccRCC [50]. As serum cholesterol decreased, *ABCG1* began to be highly expressed and promoted the efflux of cellular cholesterol to maintain serum cholesterol level.

Moreover, we found that ccRCC patients with high expression level had longer OS time than those with low expression level. It may be that the high expression of *ABCG1* can inhibit the growth of cancer cells and affect the survival of cancer cells by reducing the cholesterol content in cancer cells. Wu et al. found that liver X receptor 623 (LXR623) downregulates low-density lipoprotein receptor (LDLR) expression while upregulating *ABCA1*, leading to a decrease in intracellular cholesterol content and the occurrence of apoptosis [14]. They speculated that LXR623 could kill tumor cells by promoting cholesterol outflow [14]. *ABCG1* and *ABCA1* have many similarities: both of them are regulated by LXR623 [51–54] and they have 101 common interacting chemicals (Figure S2), promoting the outflow of cholesterol from macrophages [51–54] and regulating the expression of macrophage inflammatory cytokines [28], etc. Hence, it may be possible to kill or suppress tumor cells by upregulating *ABCG1* with LXR623.

In addition, *ABCG1* could affect tumor growth by regulating macrophages. Macrophages participate in the formation of tumor microenvironment [55,56], tumor growth, and metastasis [57–60], apoptosis [61], and play an important role in tumor immunity [62]. There are two types of it: M1 cells can produce a large number of inflammatory cytokines, which can activate the immune response and play an anti-tumor role; M2 cells promote angiogenesis, remodeling, and tumor growth [63]. In most tumor models, most of the macrophages in the tumor are shown as tumor promoting M2 phenotype [62]. Researches have shown that the deficiency of *ABCG1* increases the signaling of Toll-like receptors in macrophages, leading to an enhanced inflammatory response of macrophages to LPS or other TLR ligands [64–67], and also reduces the number and proportion of M2 phenotype [29,68]. This means that the upregulation of *ABCG1* leads to an increase in M2 macrophages, which is conducive to tumor growth. However, this is inconsistent with the result that the high *ABCG1* expression group can have a longer survival time, so more work is needed to explain the problem.

Furthermore, we identified 35 potential active drugs from 165 interplay substances with *ABCG1* in the Comparative Toxicogenomics Database (CTD) (http://ctdbase.org), of which 18 drugs can upregulate *ABCG1* expression at mRNA level, while 17 drugs can downregulate *ABCG1* expression (Table S4). In addition, six *ABCG1* target genes were identified from 60 *ABCG1* interacting genes: *ACSL4* [69], *AP1S2* [69], *ELAVL1* [70], *HNRNPL* [71], *SGO1* [69], and *UBC* [72] (Table S5).

There are still several limitations in this study. In the screening and the first rounds of validation analysis, *ABCA12* and *ABCD3* were significantly different between ccRCC and normal tissues. Unfortunately, there was no data available for *ABCA12* and *ABCD3* in the Oncomine database. But it could not exclude that *ABCA12* may be associated with the diagnosis and prognosis in ccRCC. Protein expression of *ABC* genes was analyzed before prognostic analysis in the HPA database (https://www.proteinatlas.org/). Grayscale conversion analysis was carried out on a total of 57 histological sections (11 normal tissue sections and 46 pathological sections of ccRCC patients) using ImageJ (Table S6). The t-test was then performed and a scatter plot was constructed according to the area percentage of histological sections (Figure S3). As shown in Figure S3, *ABCC3* was not statistically significant in t-test, and in the protein expression level of *ABCG1* was reduced in ccRCC patients, while positively expressed in normal tissue sections. It is not consistent with the results of mRNA level, which may be related to the type and specificity of antibody and the small sample size. Additional works and experiments need to be performed to validate them.

## Conclusion

Excluding the ABC family members that have been studied, through multiple rounds of validation, a novel diagnostic and prognostic biomarker of ccRCC – *ABCG1* – was found. According to the high expression of *ABCG1* in ccRCC and its correlation with better prognosis, it may be helpful for the diagnosis and provides new ideas for the development of molecular-targeted drugs for ccRCC.

## Supplementary Material

Supplemental Material
