# Supplementary material for: Diagnostic and prognostic value of ABC transporter family member ABCG1 gene in clear cell renal cell carcinoma
Source: Channels (Austin). 2021 Apr 7;15(1):375–85. doi: 10.1080/19336950.2021.1909301 (PMC8032227; doi:10.1080/19336950.2021.1909301)
Supplement: Supplemental Material [file KCHL_A_1909301_SM7889.rtf]

Supplemental file:
Table S1. T-test and ROC analysis of ABC transporter family members according to the GSE53757 database.
No.	Gene	Expression
(ccRCC)	Expression
(normal)	ccRCC / normal	AUC	p	
1	ABCA3	423.1	217.3	1.95	0.93	<0.0001	
2	ABCA8	143.9	567.0	0.25	0.91	<0.0001	
3	ABCA9	52.5	100.5	0.52	0.90	<0.0001	
4	ABCA12	129.5	20.9	6.20	0.91	<0.0001	
5	ABCC3	1409.0	354.8	3.97	0.96	<0.0001	
6	ABCC6	662.4	2110.3	0.31	0.94	<0.0001	
7	ABCC8	48.5	110.1	0.44	0.93	<0.0001	
8	ABCD3	2338.0	4498.0	0.52	0.95	<0.0001	
9	ABCF1	1307	1759	0.74	0.88	<0.0001	
10	ABCG1	907.4	300.1	3.02	0.97	<0.0001	


Table S2. ABCC3 expression analysis according to the Oncomine database.
Gene	Dataset	Normal
(cases)	Tumor
(cases)	Fold Change	t-test	p Value	
ABCC3
	Higgins Renal	Kidney (2)	Clear Cell Renal Cell Carcinoma (25)	5.153	10.913	8.94×10^-11^	
	Gumz Renal	Kidney (10)	Clear Cell Renal Cell Carcinoma (10)	15.080	10.509	2.38×10^-9^	
	Beroukhim Renal	Renal Cortex (10)
Renal Tissue (1)	Non-Hereditary Clear Cell Renal Cell Carcinoma (27)	4.925	8.096	1.42×10^-8^	
	Beroukhim Renal	Renal Cortex (10)
Renal Tissue (1)	Hereditary Clear Cell Renal Cell Carcinoma (32)	6.563	10.785	3.35×10^-9^	

Table S3. ABCG1 expression analysis according to the Oncomine database.
Gene	Dataset	Normal
(cases)	Tumor
(cases)	Fold change	t-test	p Value	
ABCG1	Beroukhim Renal	Renal Cortex (10)
Renal Tissue (1)	Non-Hereditary Clear Cell Renal Cell Carcinoma (27)	5.973	16.425	3.56×10^-16^	
	Beroukhim Renal	Renal Cortex (10)
Renal Tissue (1)	Hereditary Clear Cell Renal Cell Carcinoma (32)	4.918	14.673	3.12×10^-15^	
	Yusenko Renal	Fetal Kidney (2)
Kidney (3)	Clear Cell Renal Cell Carcinoma (26)	4.904	5.966	5.53×10^-4^	
	Lenburg Renal	Kidney (9)	Clear Cell Renal Cell Carcinoma (9)	2.256	3.856	0.002	
	Cutcliffe Renal	Fetal Kidney (3)
	Clear Cell Renal Cell Carcinoma (14)	4.163	7.397	7.61×10^-5^	
	Gumz Renal	Kidney (10)	Clear Cell Renal Cell Carcinoma (10)	3.454	14.013	1.66×10^-10^	


Table S4. Chemical Interactions of ABCG1 according to CTD database.
No.	Interacting Chemical	Expression
(mRNA level)	References	Organisms	
1	Cidofovir	↓	1	1	
2	Cisplatin	↓	1	1	
3	Clodronic Acid	↓	1	1	
4	Clofibrate	↓	1	1	
5	Clozapine	↑	1	1	
6	Cyclosporine	↓	1	1	
7	Cyclosporine	↑	2	1	
8	Enniatins	↑	1	1	
9	Estradiol	↓	1	1	
10	Estrogens	↓	1	1	
11	Ethinyl Estradiol	↑	2	1	
12	Fenofibrate	↑	1	1	
13	Gentamicins	↑	1	1	
14	Glucosamine	↑	1	1	
15	Ibuprofen	↑	1	1	
16	Ifosfamide	↓	1	1	
17	Ivermectin	↓	1	1	
18	Ketamine	↑	1	1	
19	Acetaminophen	↑	1	1	
20	Aetylcysteine	↓	1	1	
21	Antirheumatic Agents	↓	1	1	
22	obeticholic acid	↓	1	1	
23	Oxycodone	↓	1	1	
24	Phenytoin	↓	1	1	
25	Progesterone	↓	1	1	
26	Progesterone	↑	2	2	
27	Rifampin	↑	2	1	
28	Rosiglitazone	↑	1	1	
29	Rosuvastatin Calcium	↓	1	1	
30	Scopolamine	↑	1	1	
31	Simvastatin	↓	2	2	
32	Sunitinib	↑	1	1	
33	Telmisartan	↑	1	1	
34	Tretinoin	↑	2	1	
35	Troglitazone	↑	1	1	

Table S5. Target genes of ABCG1 according to CTD database.
No.	Source Gene	Target Gene	Source Organism	Target Organism	Assay	Interaction Type	Throughput	Reference	
1	ACSL4	ABCG1	Homo sapiens	Homo sapiens	Affinity Capture-MS	physical	high	Hein MY, et al. (2015).	
2	AP1S2	ABCG1	Homo sapiens	Homo sapiens	Affinity Capture-MS	physical	high	Hein MY, et al. (2015).	
3	ELAVL1	ABCG1	Homo sapiens	Homo sapiens	Affinity Capture-RNA	physical	high	Abdelmohsen K, et al. (2009).	
4	HNRNPL	ABCG1	Homo sapiens	Homo sapiens	Affinity Capture-RNA	physical	high	Fei T, et al. (2017).	
5	SGO1	ABCG1	Mus musculus	Homo sapiens	Affinity Capture-MS	physical	high	Hein MY, et al. (2015).	
6	UBC	ABCG1	Rattus norvegicus	Rattus norvegicus	Affinity Capture-MS	physical	low	Na CH, et al. (2012).	


Table S6. Area percentage of histological sections in Grayscale conversion analysis.
NO.	ABCC3		ABCG1	
	KIRC	Normal		KIRC	Normal	
1	32.12	47.48		37.11	39.01	
2	31.77	29.60		26.92	40.54	
3	24.89	26.33		25.69	39.97	
4	18.85	26.22		25.81	37.00	
5	26.06	27.55		22.58	37.99	
6	24.67			22.79	36.89	
7	23.15			33.03		
8	59.79			43.04		
9	35.17			32.32		
10	22.96			20.26		
11	27.98			25.89		
12	30.08			34.44		
13	28.02			21.95		
14	21.46			19.14		
15	34.19			23.16		
16	25.09			20.31		
17	21.05			17.47		
18	33.22			28.55		
19	31.05			33.77		
20	37.53			36.35		
21	19.03			21.07		
22	17.95			23.08		
23	32.20			25.72		


Figure S1. The mRNA expression of ABCC3, ABCF1 and ABCG1 in different types of cancers by Oncomine analysis. The number in the colored cell represents the number of analyses meeting thresholds. Cell color is determined by the gene rank. The more intense red (over-expression) or blue (under-expression) indicates a more highly significant over-expressed or under-expressed gene.


Figure S2. (A) 20 genes had comparable sets of interacting chemicals to ABCG1. (B) Analysis of curated chemicals according to CTD database. All chemicals related to ABCG1(165), All chemicals related to ABCA1(300), Chemicals common to ABCG1 and ABCA1(101).

Figure S3. Scatter plots of protein expression of ABCC3 and ABCG1 according to the HPA database. The black "***" represents p <0.001.
